# Short-Term Depression of Axonal Spikes at the Mouse Hippocampal Mossy Fibers and Sodium Channel-Dependent Modulation

**DOI:** 10.1523/ENEURO.0415-17.2018

**Published:** 2018-02-20

**Authors:** Shunsuke Ohura, Haruyuki Kamiya

**Affiliations:** 1 Department of Neurobiology, Hokkaido University Graduate School of Medicine, Sapporo 060-8638, Japan

**Keywords:** action potential, axon, hippocampus, mossy fiber, short-term plasticity

## Abstract

Axonal spike is an important upstream process of transmitter release, which directly impacts on release probability from the presynaptic terminals. Despite the functional significance, possible activity-dependent modulation of axonal spikes has not been studied extensively, partly due to inaccessibility of the small structures of axons for electrophysiological recordings. In this study, we tested the possibility of use-dependent changes in axonal spikes at the hippocampal mossy fibers, where direct recordings from the axon terminals are readily feasible. Hippocampal slices were made from mice of either sex, and loose-patch clamp recordings were obtained from the visually identified giant mossy fiber boutons located in the stratum lucidum of the CA3 region. Stimulation of the granule cell layer of the dentate gyrus elicited axonal spikes at the single bouton which occurred in all or none fashion. Unexpected from the digital nature of spike signaling, the peak amplitude of the second spikes in response to paired stimuli at a 50-ms interval was slightly but reproducibly smaller than the first spikes. Repetitive stimuli at 20 or 100 Hz also caused progressive use-dependent depression during the train. Notably, veratridine, an inhibitor of inactivation of sodium channels, significantly accelerated the depression with minimal effect on the initial spikes. These results suggest that sodium channels contribute to use-dependent depression of axonal spikes at the hippocampal mossy fibers, possibly by shaping the afterdepolarization (ADP) following axonal spikes. Prolonged depolarization during ADP may inactivate a fraction of sodium channels and thereby suppresses the subsequent spikes at the hippocampal mossy fibers.

## Visual Abstract

**Figure:**
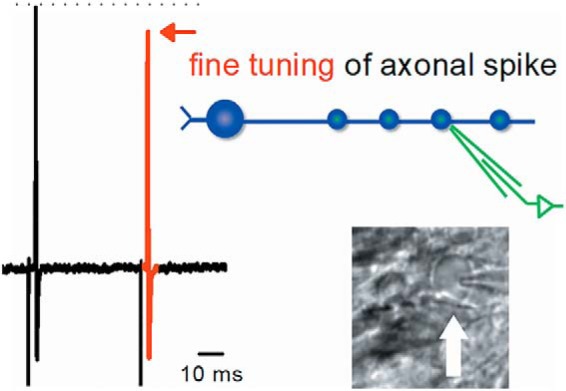

## Significance Statement

Spike signaling along axons is thought to highly reliable digital process. In this study, we tested the possibility of analog tuning of axonal spikes using direct recordings from single hippocampal mossy fiber terminals. We found that axonal spikes are subject to robust use-dependent short-term depression. Notably, the application of veratridine, an inhibitor of inactivation of sodium channels, selectively accelerates short-term depression with minimal effect on the initial axonal spikes. These results illustrate the novel form of short-term plasticity of axonal spikes in single axon terminal levels, and suggest that slow activating sodium channels of persistent type (I_NaP_) or resurgent types (I_NaR_), different from the transient type (I_NaT_) responsible for spike generation, might be involved in modulation of paired-pulse depression (PPD) of axonal spikes.

## Introduction

Spike propagation along axon is highly reliable digital processes to carry neuronal information for a long distance without attenuation (Hodgkin and Huxley, 1952; Debanne, 2004; Kole and Stuart, 2012). Recent studies, however, have suggested that axonal spikes are regulated by the preceding neuronal activity or by the subtle changes in the local microenvironment due to influence from surrounding neuron and glia (Dugladze et al., 2012; Ruiz and Kullmann, 2012; Debanne et al., 2013; Kawaguchi and Sakaba, 2015). Frequency-dependent refractoriness at short intervals is well known examples of use-dependent regulation of axonal spikes due to intrinsic kinetic properties of ionic channels in axonal membrane. In addition, their excitability is also shown to be affected by transmitters released from the surrounding neuron and glia (Kamiya et al., 2002; Alle and Geiger, 2007; Sasaki et al., 2011; Uchida et al., 2012). Since hippocampal mossy fibers are unmyelinated axons readily accessible by these neuro- or glio-transmitters, and are suggested to express GABA_A_, glycine, and kainate-type glutamate receptors on the axonal membranes (Kamiya and Ozawa, 2000; Schmitz et al., 2001; Ruiz et al., 2003; Kubota et al., 2010), it was expected that activation of these axonal receptors may also contribute to regulate action potential conduction and synaptic transmission at the mossy fiber-CA3 synapses.

Because of the reasonable size for direct electrophysiological recordings from the large boutons of typically 3–5 μm in diameter (Geiger et al., 2002; Bischofberger et al., 2006), hippocampal mossy fiber is studied intensively not only for synaptic but also for axonal mechanisms. The new findings obtained using the direct recording from the boutons include spike broadening during repetitive stimuli (Geiger and Jonas, 2000), spike amplification at *en passant* boutons (Engel and Jonas, 2005), passive electrical signaling along the axon (Alle and Geiger, 2006), spike initiation at the proximal axons (Schmidt-Hieber et al., 2008), and energy-efficient spike generation (Alle et al., 2009).

On the other hand, activity-dependent modification of axonal spikes has not studied extensively so far, except for use-dependent spike broadening of action potentials during repetitive stimuli (Geiger and Jonas, 2000). They showed robust use-dependent broadening of axonal spikes recorded from mossy fiber terminals, and suggested that accumulated inactivation of voltage-gated K^+^ channels underlies this unique form of short-term plasticity.

In the present study, we examined the possible use-dependent modification of spike signaling along hippocampal mossy fibers, unmyelinated axons with *en passant* structures typical for cortical axons. We also explored the mechanisms underlying this novel form of short-term plasticity, and addressed the possible contribution of afterdepolarization (ADP), a hallmark of axonal spikes. Since ADP following axonal spike is reported to be mediated by activation of resurgent-type Na^+^ current at the calyx of Held presynaptic terminals (Kim et al., 2010) and is robustly enhanced by veratridine, an inhibitor of inactivation of Na^+^ channel, we tested if veratridine modulates use-dependent depression of axonal spikes. Prominent use-dependent effect of veratridine suggests that sodium channels play important roles not only in generation of axonal action potentials, but also in modulating short-term plasticity by affecting ADP following axonal action potentials.

## Materials and Methods

### Animals

C57BL/6J mice were initially purchased (Japan SLC) and later bred in-house. All animal procedures were performed in accordance with the Hokkaido University animal care committee's regulations. Every effort to minimize suffering and the numbers of animals was made throughout the study.

### Preparation of hippocampal slices

Transverse hippocampal slices of 300 μm thick were prepared from C57BL/6J mice of either sex (p14–p43, number of animals = 58) as described previously (Shimizu et al., 2008) with some modifications. Animals were anesthetized with ether and the brain was dissected out in an ice-cold sucrose solution containing the following: 40 mM NaCl, 25 mM NaHCO_3_, 10 mM glucose, 150 mM sucrose, 4 mM KCl, 1.25 mM NaH_2_PO_4_, 0.5 mM CaCl_2_, and 7 mM MgSO_4_ (Geiger et al., 2002). Transverse hippocampal slices were cut using a VT1200S microslicer (Leica Biosystems), and the above solution was replaced with a NMDG-HEPES recovery solution containing the following: 93 mM NMDG, 30 mM NaHCO_3_, 25 mM glucose, 20 mM HEPES, 2.5 mM KCl, 1.2 mM NaH_2_PO_4_, 5 mM Na-ascorbate, 2 mM thiourea, 3 mM Na-pyruvate, 0.5 mM CaCl_2_, and 10 mM MgSO_4_ and incubated for no longer than 15 min (Ting et al., 2014). Then, the solution was exchanged again with artificial CSF (ACSF) containing the following: 127 mM NaCl, 1.5 mM KCl, 1.2 mM KH_2_PO_4_, 26 mM NaHCO_3_, 10 mM glucose, 2.4 mM CaCl_2_, and 1.3 mM MgSO_4_, and the slices were kept in an interface-type chamber saturated with 95% O_2_ and 5% CO_2_ at room temperature (∼25°C).

### Electrophysiology

**Figure 1.**
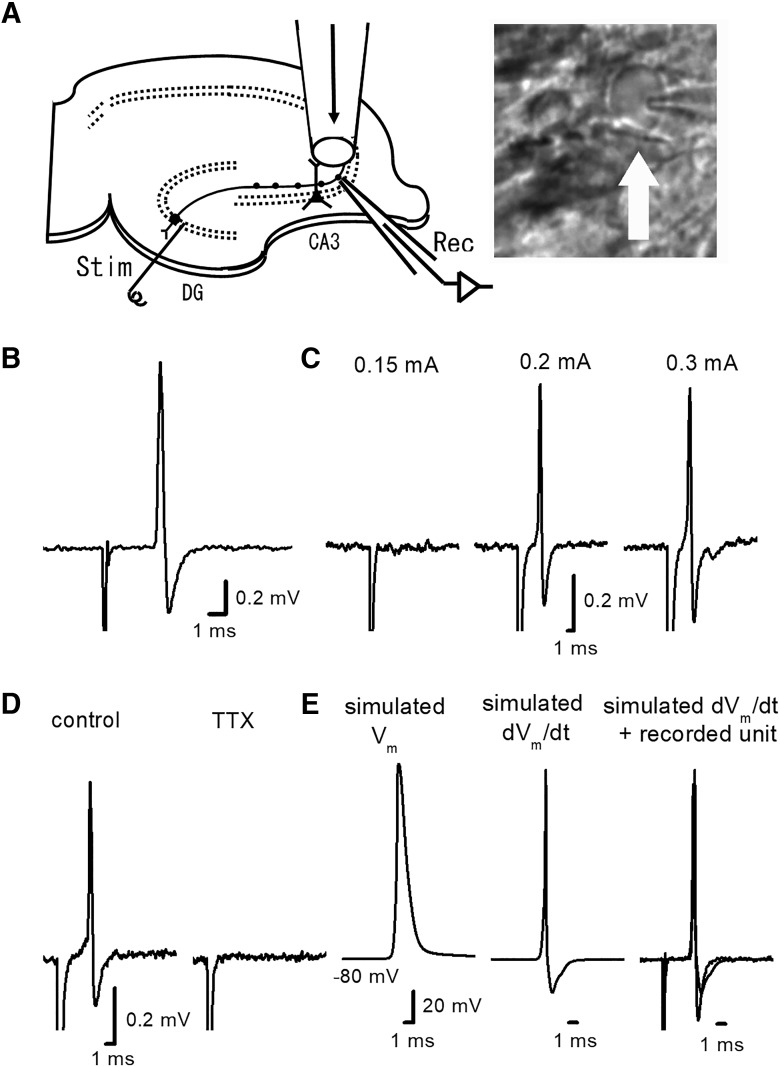
Loose-patch clamp recordings of axonal spikes from the single mossy fiber boutons. ***A***, Schematic drawing of experimental arrangement. Stimulating electrode was placed in the granule cell layer of the dentate gyrus, and the evoked responses were recorded from visually-identified single mossy fiber boutons. Surrounding region of the recording site was focally perfused with a continuous flow of perfusate through a flow-pipe. A photograph showing IR-DIC image of the recorded bouton (arrow). ***B***, Representative traces of the axonal spikes recorded from the single mossy fiber boutons. ***C***, All-or-none feature of the axonal spikes, which appear above the threshold stimulus intensity (0.2 mA in this recording). ***D***, Effect of focal application of TTX at 0.5 μM. ***E***, Comparison of the time course of simulated dV_m_/dt and the recorded axonal spikes. Simulated membrane potential (V_m_) during axonal action potential was calculated according to the latest model of action potentials at mossy fibers (see Materials and Methods). In the right panel, the recorded axonal spike in ***B*** was superimposed with the first derivative of simulated V_m_ (dV_m_/dt, middle panel).

The slices were perfused with the Ca^2+^-free ACSF (equal concentration of Mg^2+^ was replaced for Ca^2+^; 0 CaCl_2_ and 3.7 MgSO_4_) at ∼2 ml/min and maintained at 24–26°C in a recording chamber. In addition, the slice surface of the recording site was locally perfused with the above solution at ∼0.2 ml/min through a flow pipe with a 250-μm open-tip diameter connected to an electromagnetic valve system (Valve Bank; Automate Scientific) for faster exchange of solution selectively around the recording sites (Fig. 1*A*). The Ca^2+^-free ACSF was used to suppress all synaptic transmission and therefore eliminate possible recording from postsynaptic neurons.

For extracellular recording of axonal spikes from single mossy fiber boutons, glass pipettes containing the recording solution (typically 3–6 MΩ electrode resistance) were placed on the visually-identified putative boutons in the stratum lucidum under IR-DIC microscope (BX51WI, Olympus), and gentle suction was applied to the recording pipettes. Loose patch configuration was used to achieve less-invasive recording from the small boutons for a long period. For instance, even under continuous focal perfusion around the recoding site (see above; Fig. 1*A*), stable recordings for long periods up to several hours are readily feasible, and therefore are suited for quantitative pharmacological study of bath or focally applied drugs.

In experiments shown in Figure 7, whole-cell current clamp recordings of action potentials and ADP from the granule cell soma (Kamiya and Ozawa, 2000) were performed. Patch pipettes were filled with an internal solution (pH 7.3) containing: 140 mM potassium gluconate, 20 mM KCl, 0.2 mM EGTA, 2.0 mM MgCl_2_, 10 mM HEPES, and 2.0 mM Mg-ATP. The resistance of the pipette was 4–8 MΩ when filled with the internal solution. The access resistance was typically 15–30 MΩ immediately after obtaining whole-cell recordings, and was not allowed to vary by >20% during the course of the experiment.

**Figure 2.**
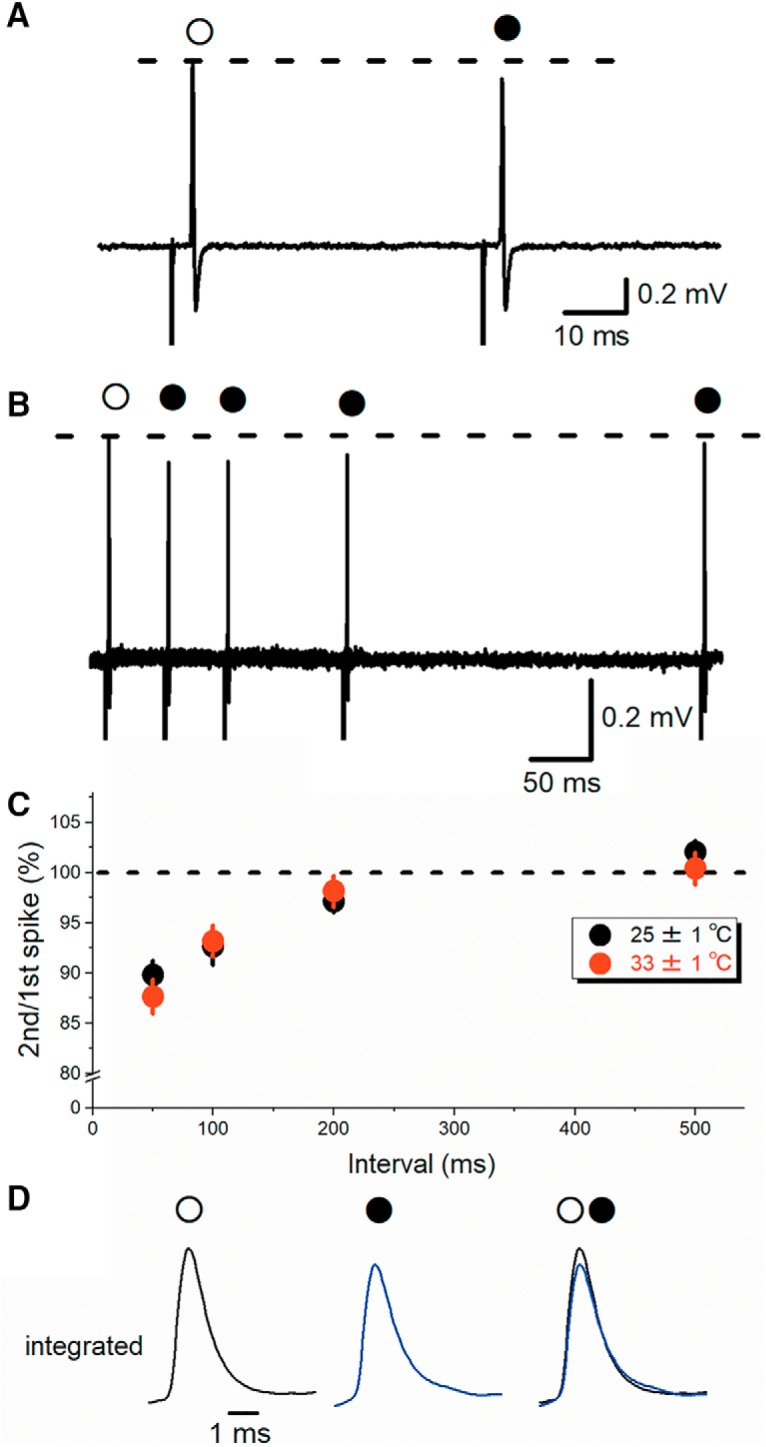
PPD of axonal spikes recorded from single mossy fiber boutons. ***A***, At a 50-ms interval, the amplitude of the second spike (closed circle) was slightly reduced than the first spike (open circle). ***B***, Superimposed traces of paired-pulse responses at 50-, 100-, 200-, and 500-ms intervals. ***C***, Time course of PPD of axonal spikes recorded at 25 ± 1°C are shown in black circles (*n* = 9). Data of similar experiments recorded at 33 ± 1°C are also shown in red circles (*n* = 7). ***D***, Time-integrated traces of axonal spikes recorded extracellularly by loose-patch clamp configuration, which are supposed to reflect intracellular membrane potential changes during action potentials, in response to the first (open circle) and second stimuli (closed circle, blue trace). Note that superimposed traces in the right panel show reduction of the peak amplitudes.

All recordings were made at room temperature (25 ± 1°C), except in the experiments at more physiologic temperatures (33 ± 1°C) shown in red circles in Figure 2*C*. Extracellular axonal spikes at mossy fiber boutons or intracellular action potentials at the granule cell soma were recorded with glass pipettes using a Multiclamp 700B amplifier (Molecular Devices). Signals were filtered at 10 kHz with 4-pole Bessel filter, sampled at 20 kHz, and analyzed offline with pCLAMP10 software (Molecular Devices).

### Simulation

Simulated membrane potential (V_m_) during axonal action potential at the hippocampal mossy fibers was calculated according to the Schmidt-Hieber’s model assuming “eight states” gating of sodium channels (accession: 128079; https://senselab.med.yale.edu/ModelDB/). This model is the latest revision of the mossy fiber model which is optimized for electrophysiological data obtained by direct recording from axonal blebs (Schmidt-Hieber and Bischofberger, 2010). In this study, we conducted a simulation to validate the faithful monitoring of axonal spikes using this latest model.

### Chemicals

Veratridine was purchased from Sigma-Aldrich. Tetrodotoxin (TTX) was purchased from Funakoshi. All other chemicals were purchased from Wako Pure Chemical Industries.

### Statistics

Data are expressed as the mean ± SEM, and *n* represents the number of recording boutons. Statistical analysis for comparison between the two paired groups were performed by Wilcoxon signed-rank test, and *p* < 0.05 was accepted for significance. All statistical analyses were performed using R software (version 3.4.1)

## Results

### Recording of axonal spikes from single mossy fiber boutons

Axonal spikes elicited by stimulation of granule cell were directly recorded from a visually-identified mossy fiber boutons in mouse hippocampal slices (Fig. 1*A*). Electrical stimuli at the granule cell layer of dentate gyrus evoked the bi-phasic responses at the recorded boutons (Fig. 1*B*). These responses are likely to represent action potentials propagated along the mossy fiber axons, from the reasons mentioned below. First, these responses were evoked in all or none fashion in response to threshold stimuli (Fig. 1*C*). Second, focal application (Kamiya, 2012) of low concentration of TTX (0.5 μM) abolished the responses (Fig. 1*D*), supported by the high level expression of voltage-dependent Na^+^ channels at the mossy fiber boutons (Engel and Jonas, 2005).

It has been suggested that action potentials recorded extracellularly by loose-patch configuration well match with that of the first derivative of membrane potential (Meeks et al., 2005). To check for the adequate monitoring of axonal spikes in these recording conditions, we compared the time course of the recorded axonal spike with that of the first derivative of simulated action potentials at the mossy fibers (Fig. 1*E*). For this purpose, we adopted the model assuming eight states gating of sodium channels at the hippocampal mossy fibers (Schmidt-Hieber and Bischofberger, 2010). The time course of the recorded axonal spike is almost proportional to that of the first derivative of simulated action potential (dV_m_/dt). All these suggested that the recorded responses are likely to represent adequately monitored axonal spikes from the single mossy fiber boutons.

### Paired-pulse depression (PPD) of axonal spikes

Then, we examined activity-dependent modulation of the axonal spikes. In response to paired stimuli at a 50-ms interval, the peak amplitudes of second spikes were slightly, but reproducibly smaller than first spikes (Fig. 2*A*; 89.3 ± 0.9% of control, *n* = 29, *p* = 0.0000027). To explore the time course of PPD of axonal spikes, interstimulus intervals were varied from 50 to 500 ms (Fig. 2*B*). Depression were prominent at short intervals and almost recovered at 500-ms intervals (Fig. 2*C*; 89.8 ± 1.5%, 92.6 ± 1.8%, 97.1 ± 0.9%, and 102.1 ± 1.1% of control at 50-, 100-, 200-, and 500-ms intervals, respectively; *n* = 9). These results showed that the PPD lasts for several hundreds of ms.

Recordings at room temperature may slow down channel gating and thereby affect the degree and the time course of depression of axonal spikes. To test for depression of axonal spikes at more physiologic recording temperature, similar experiments were conducted at 33 ± 1°C. Substantial depression still remained at intervals shorter than 200 ms (Fig. 2*C*, red circles; 87.7 ± 1.7%, 93.2 ± 1.5%, 98.2 ± 1.6%, and 100.4 ± 1.6% of control at 50-, 100-, 200-, and 500-ms intervals, respectively; *n* = 7). These results showed that the PPD occurs even at the physiologic temperature.

Since the time course of the recorded axonal spike was expected to be proportional to that of the first derivative of action potentials recorded intracellularly, we reconstituted time course of membrane potential transients by calculating the time-integral of axonal spikes recorded extracellularly. As expected from inactivation of sodium channels by prolonged depolarization during ADP, the peak amplitudes of time-integrals of axonal spikes were reduced in responses to paired stimuli at 50-ms intervals (to 94.4 ± 3.4%, *n* = 20, *p* = 0.01531; Fig. 2*D*). We also measured half-width of time-integrals of the extracellularly recorded axonal spike, although the difference was statistically not significant (to 104.2 ± 12.6%, *n* = 20, *p* = 0.7012).

**Figure 3.**
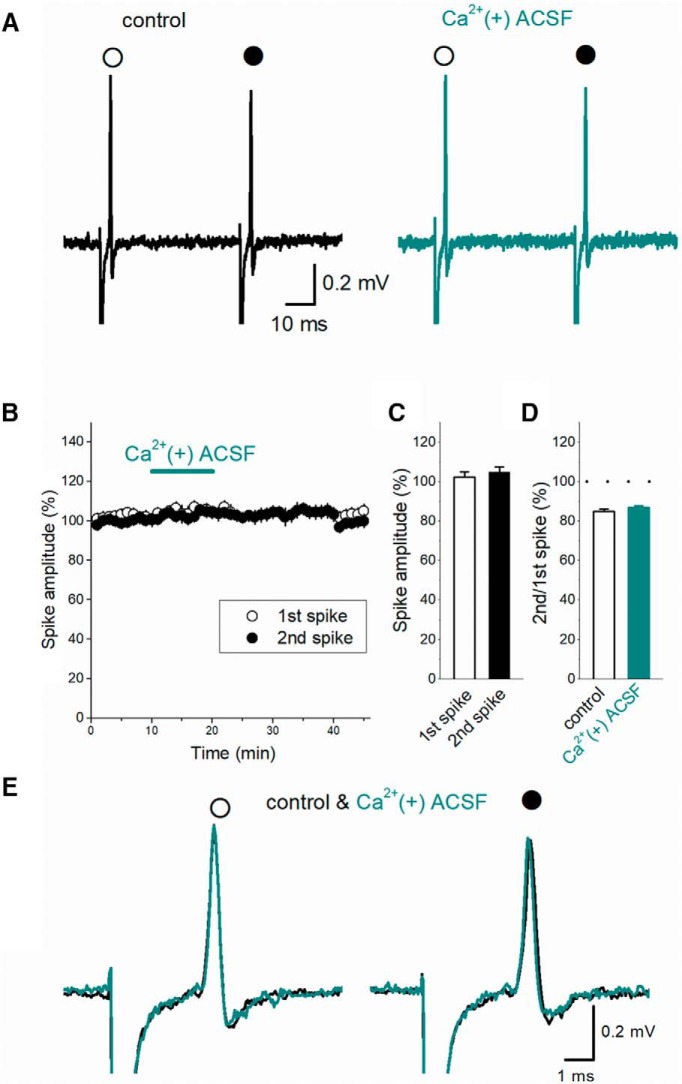
Minimal effects of Ca^2+^-containing [Ca^2+^(+)] ACSF on axonal spikes. ***A***, Focal application of Ca^2+^(+) ACSF to the surrounding area of the recorded boutons (see Fig. 1*A*) exhibited no clear effects on the 1st (open circles) and 2nd (closed circles) axonal spikes. ***B***, Time course of the amplitude of the first and second spikes during Ca^2+^(+) ACSF application. ***C***, Summary data of the effect of Ca^2+^(+) ACSF on the first (open bar) and second spikes (closed bar, *n* = 8). ***D***, Summary data of PPD of axonal spikes in control condition (open bar) and in Ca^2+^(+) ACSF. ***E***, Superimposed traces of the first (open circle) and 2nd spike (closed circle) in the control condition and in Ca^2+^(+) ACSF.

To confirm spike recordings from axon terminals, we used Ca^2+^-free solution to exclude the possibility of synaptically evoked spikes in postsynaptic cells. One may argue that recordings in Ca^2+^-free conditions may perturb observation in physiologic conditions. To check for the roles of physiologic concentration of Ca^2+^, we examined the effect of application of Ca^2+^-containing ACSF after establishment of recording in Ca^2+^-free solution. PPD of axonal spikes was almost unaffected by application of Ca^2+^-containing solution (Fig. 3*A*). The first (open circles) as well as second spikes (closed circles) were almost unchanged (to 102.3 ± 2.5% and 104.7 ± 2.8% of control, *n* = 8; Fig. 3*B*,*C*). The effects of Ca^2+^-containing solution on the first and the second responses were statistically not different (*p* = 0.7422 and *p* = 0.4609, respectively). The wave form of the first and second axonal spikes were almost unchanged by Ca^2+^-containing solution (Fig. 3*E*). These results indicate that PPD of axonal spikes occurs in physiologic condition.

### Suppression of PPD by veratridine

**Figure 4.**
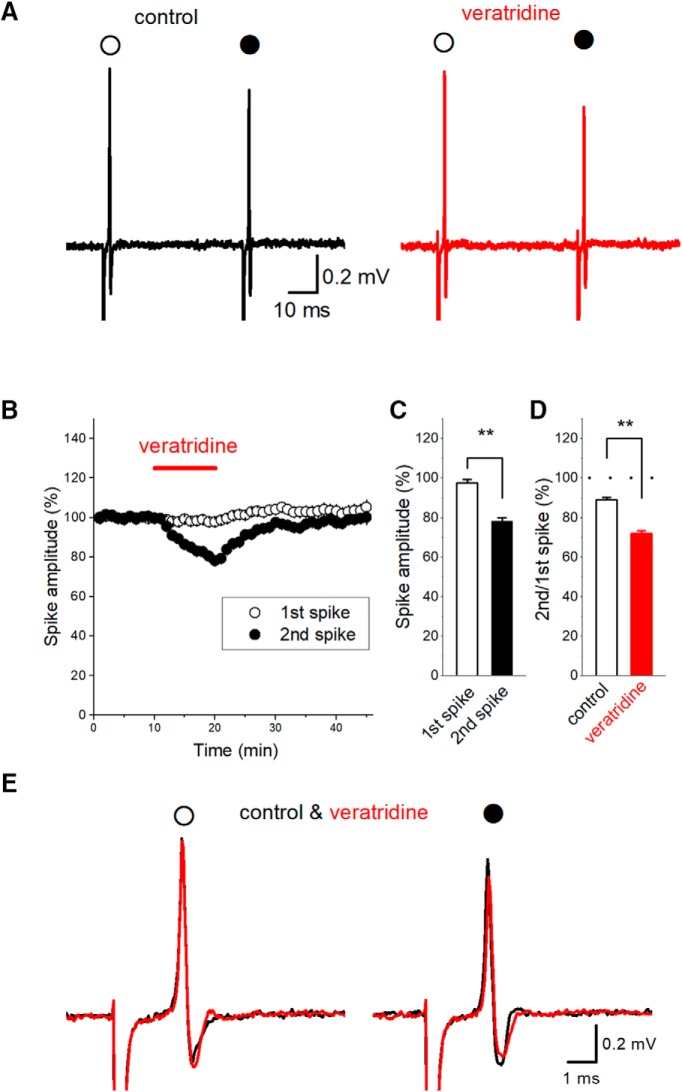
Selective modulation of PPD of axonal spikes by veratridine, an inhibitor of inactivation of sodium channels. ***A***, Focal application of 1 μM veratridine selectively suppressed the amplitude of the second spike (closed circle) with minimal effect on the first spike (open circle). ***B***, Time course of the amplitude of the first and second spikes during veratridine application (*n* = 20). ***C***, Summary data on the effect of veratridine on the first (open bar) and second spikes (closed bar, *n* = 20, ** *P* < 0.01). ***D***, Summary data of PPD of axonal spikes in control condition (open bar) and in the presence of veratridine (closed bar). ***E***, Superimposed traces of the first (open circle) and 2nd spike (closed circle) in control condition and in the presence of veratridine.

Since the time course of PPD, as shown in Figure 2, is similar to that of ADP, which typically follows action potential of hippocampal mossy fibers (Geiger and Jonas, 2000; Kamiya et al., 2002), we supposed that PPD are caused by progressive inactivation of voltage-gated Na^+^ channels (He et al., 2002) due to prolonged ADP. To test this, veratridine, an inhibitor of inactivation of Na^+^ channels, was used, since the previous study at the calyx of Held axon terminals showed that veratridine robustly enhances ADP (Kim et al., 2010). If ADP was partly mediated by slowly activating Na^+^ channels, possibly either persistent type I_NaP_ (D'Ascenzo et al., 2009; Kole, 2011; Ghitani et al., 2016) or resurgent type I_NaR_ (Raman and Bean, 1997), prolonged ADP may inactivate significant fraction of transient Na^+^ channels I_NaT_ responsible for generation of action potentials in axons. As expected, focal application of 1 μM veratridine notably reduced the peak amplitude of the second spikes in responses to the paired-stimuli at a 50-ms interval, with minimal effects on the first responses (Fig. 4*A*). The second spikes (closed circles) were reduced to 78.1 ± 1.8% of control, whereas the first responses (open circles) were almost unchanged (to 97.6 ± 1.8% of control, *n* = 20; Fig. 4*B*,*C*). The effect of veratridine on the first and the second responses was statistically significant (*p* = 0.00000191). As a consequence, PPD of axonal spikes at a 50-ms interval was enhanced by 1 μM veratridine (89.0 ± 1.1% in control and 71.9 ± 1.5% during veratridine application, *n* = 20, *p* = 0.000001907; Fig. 4*D*). This use-dependent depression of axonal spikes by veratridine suggests the notion that ADP are partly mediated by some types of sodium channels with slow activation such as resurgent-type I_NaR_ or persistent-type I_NaP_. Prolonged depolarization during ADP by activation of these slow activating sodium channels may inactivate a fraction of transient sodium channels I_NaT_ responsible for generation of action potentials, and thereby suppress the second spikes. Sustained depolarization at the time of the second stimulus were also supported by the finding that the latency of the second spike was shorter than the first spike (to 95.2 ± 0.9%, *n* = 20, *p* = 0.000558). This also suggests that ADP plays a regulatory role on the second spikes in response to paired-stimuli.

### Veratridine accelerates depression of axonal spikes by repetitive stimuli

**Figure 5.**
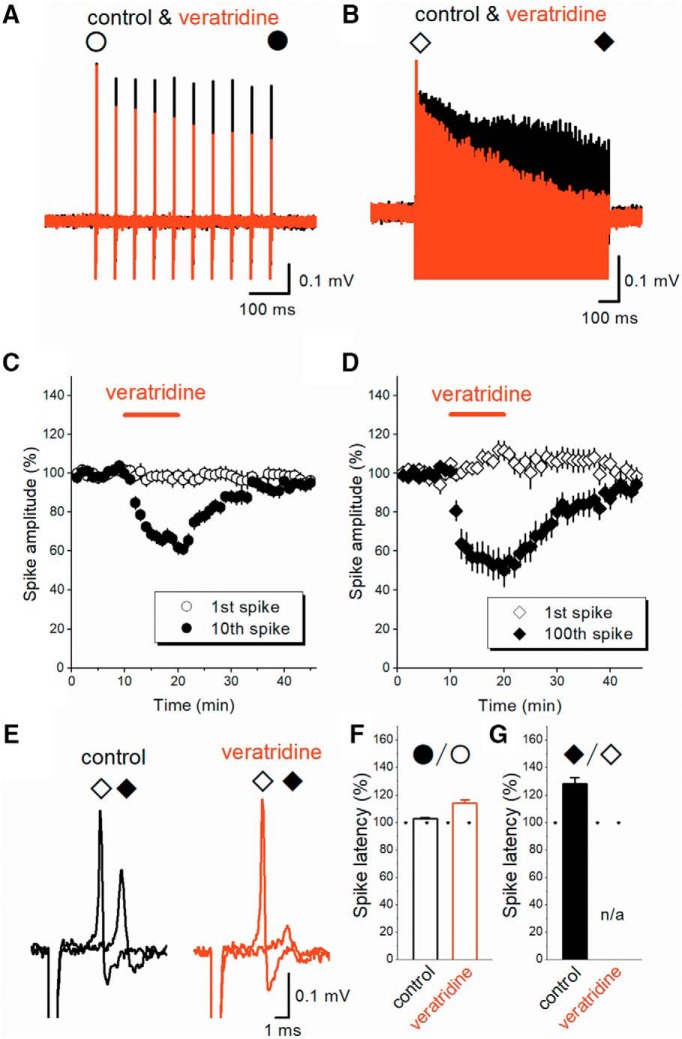
Veratridine-induced use-dependent modulation of axonal spikes during repetitive stimuli. ***A***, ***B***, Veratridine at 1 μM minimally affected the first spike (open circle), while selectively reduced the subsequent responses to 20 Hz (***A***) and 100 Hz (***B***) trains. ***C***, ***D***, Time course of the first, 10th or 100th spikes during veratridine application (*n* = 13 and 14, respectively). ***E***, Superimposed traces of the first (open diamond) and 100th spike during 100-Hz train (closed diamond) in the control condition and in the presence of veratridine. ***F***, ***G***, Summary data of latency of the first and the last spikes during 20-Hz (***F***) and 100-Hz trains (***G***). The 100th spikes during 100-Hz trains in the presence of veratridine (***G***) were reduced in size substantially, and therefore were unable to measure the latency quantitatively.

We also examined the effect of 1 μM veratridine on the responses to repetitive stimuli. First, we applied 20-Hz stimuli for 10 times, same 50-ms interval adopted for paired-pulse protocol in Figure 4, to see whether the effect is cumulative on multiple stimuli. Veratridine caused the progressive decrease of the peak amplitude of axonal spikes (Fig. 5*A*) with minimal effect on the first responses. We also tested the effect of veratridine on the responses to high-frequency stimulation at 100 Hz for 1 s. Although axonal spikes faithfully followed in response to almost all stimuli even at 100 Hz, the amplitudes declined gradually during the train (Fig. 5*B*). Application of 1 μM veratridine again selectively suppressed the later responses with minimal effect on the first responses.

The 10th spikes during 20-Hz trains (closed circles) were reduced to 61.8 ± 3.1%, whereas the first responses (open circles) were 98.8 ± 3.2% of control (Fig. 5*C*; *n* = 13). The effects of veratridine on the 10th responses were different from those on the first spikes (*p* = 0.000244). The 100th spikes during 100-Hz trains (open circles) were reduced to 50.2 ± 8.2%, whereas the first responses (closed circles) were 111.6 ± 4.8% of control (Fig. 5*D*; *n* = 14). The effect of veratridine on the first and the 100th responses was statistically significant (*p* = 0.000366). These observations are consistent with the prediction of inactivation of transient Na^+^ channels I_NaT_ by enhanced ADP due to application of veratridine.

In line with inactivation of I_NaT_, not only amplitude but also latency and duration of axonal spikes were prolonged during the train (Fig. 5*E*). The latency to peak of the 10th spike during 20-Hz train was prolonged relative to the 1st spike (to 102.6 ± 1.1%, *n* = 13, *p* = 0.0002441) and further delayed by veratridine (to 114.1 ± 2.4%, *n* = 13, *p* = 0.0004883; Fig. 5*F*). Veratridine also prolonged the duration of 10th spikes (151.2 ± 10.8%) with minimal effect on the first spikes (97.8 ± 2.1%, *n* = 13). The latency to peak of the 100th spike during 100-Hz train was prolonged compared to the 1st spike (to 128.2 ± 4.5%, *n* = 14, *p* = 0.00109; Fig. 5*G*). These findings also support the notions that cumulative inactivation of sodium channels underlies use-dependent depression of axonal spikes.

### TTX partly restores PPD of axonal spikes

**Figure 6.**
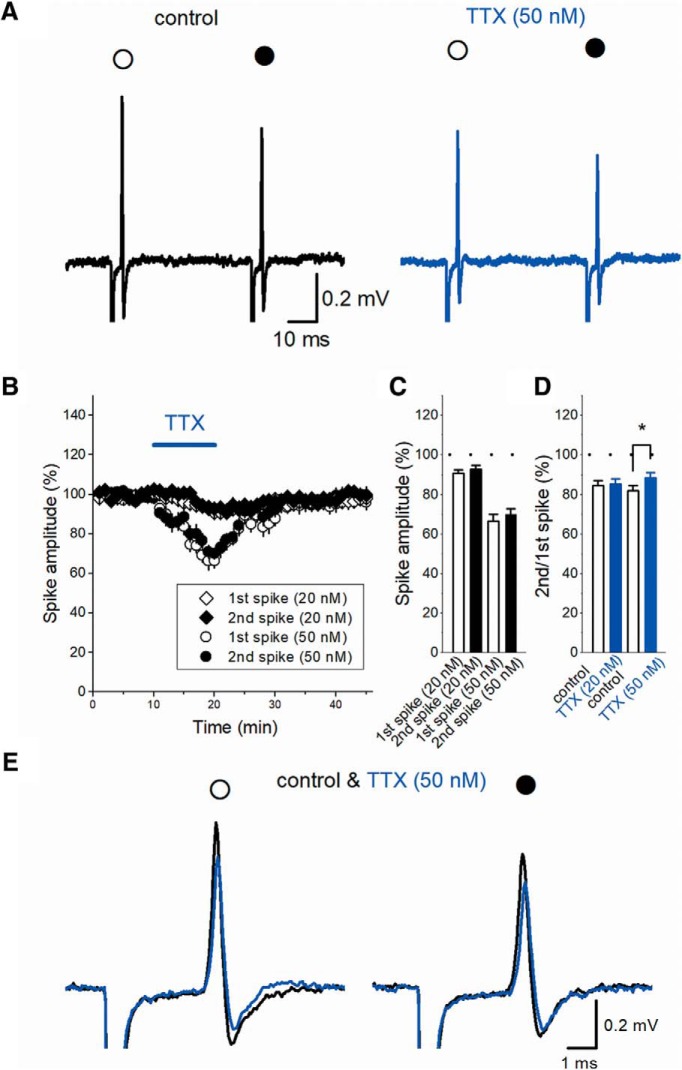
Effect of low concentration of TTX on axonal spike. ***A***, TTX at 50 nM partially suppressed both the first (open circle) and second spike (closed circle). ***B***, Time course of the amplitude of the first and 2nd spikes during application of 20 nM and 50 nM TTX (*n* = 7 and 9, respectively) as shown by open and closed diamonds and circles, respectively. ***C***, Summary data of the effect of 20 and 50 nM TTX on the first (open bar) and second spikes (closed bar). ***D***, PPD was weakly restored by 50 nM TTX (closed bar, *n* = 9, *p < 0.05), while 20 nM TTX did not affect significantly (*n* = 7). ***E***, Superimposed traces of the first (open circle) and second spike (closed circle) in control condition and in the presence of 50 nM TTX.

To further get insights into the activity-dependent tuning of the axonal spikes, we also tested the effect of a sodium channel blocker TTX, since low concentration of TTX has been shown to suppress ADP at the axon terminals of calyx of Held (Kim et al., 2010). Application of 50 nM TTX suppressed the first and second spikes (Fig. 6*A*) to 66.4 ± 3.6% and 70.1 ± 2.7% (*n* = 9; Fig. 6*B*,*C*). Application of 20 nM TTX weakly suppressed the first and second spikes to 90.8 ± 1.7% and 92.8 ±1.9% (*n* = 7; Fig. 6*B*,*C*). In line with the notion that ADP may underlie depression of subsequent axonal spikes, 50 nM TTX weakly, but significantly, restored PPD of axonal spikes (82.3 ± 2.5% in control and 88.6 ± 2.5% during TTX application, *n* = 9, *p* = 0.03906; Fig. 6*D*).

### Veratridine enhances ADP in granule cell soma

**Figure 7.**
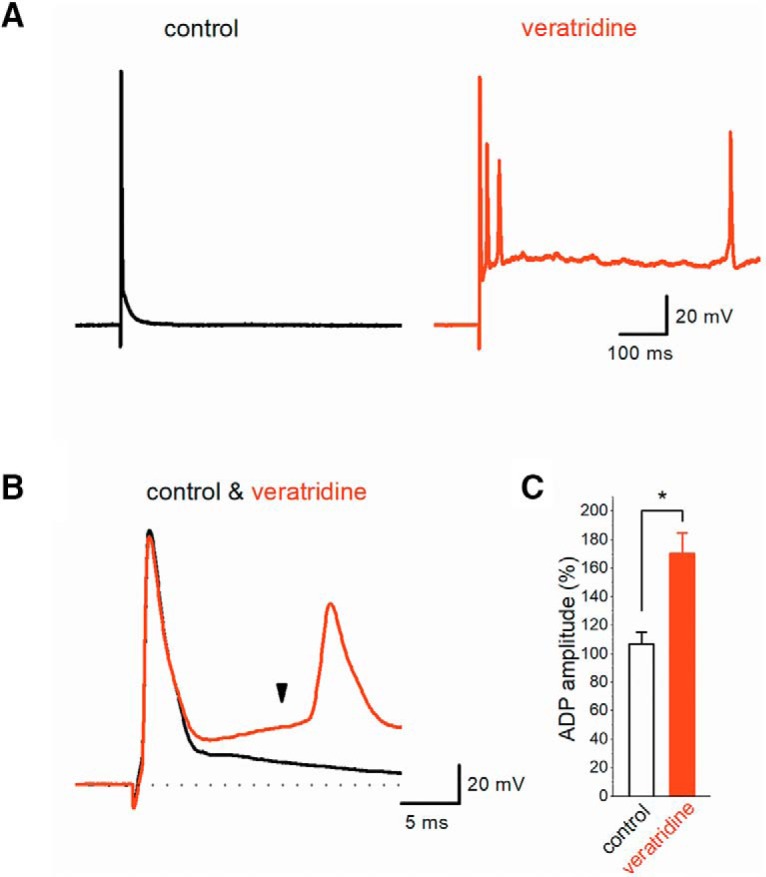
Veratridine potentiated ADP in the granule cell soma. ***A***, Veratridine at 1 μM strongly enhanced ADP following action potentials elicited by brief current injection into the dentate granule cells, which originates the mossy fiber axons. Prolonged ADP during veratridine application was often accompanied by multiple spikes after current injection. ***B***, Expanded time course of action potential and ADP. ADP amplitude was quantified at 10 ms after the peak of the action potential (arrowhead). ***C***, Summary data on the effect of veratridine on the ADP amplitude (*n* = 7, *p < 0.05).

The results so far support the notion that ADP-enhancement by veratridine may cause much inactivation of I_NaT_ and thereby suppresses subsequent axonal spikes in use-dependent manner. To further test this, we recorded action potentials from the granule cell soma in the dentate gyrus, which originates mossy fiber axons and exhibits pronounced ADP following action potentials as in mossy fiber terminals (Geiger and Jonas, 2000). Brief current injection (500–1000 pA, 1 ms) elicited action potentials followed by ADP (Fig. 7*A*). Application of 1 μM veratridine robustly enhanced and prolonged ADP (Fig. 7*A–C*) and caused multiple spiking by single stimuli, as similar to the calyx of Held axon terminals (Kim et al., 2010). Application of 1 μM veratridine enhanced the amplitude of ADP, as measured at 10 ms after the peak of action potentials (Fig. 7*B*), to 170.1 ± 14.7% (*n* = 7, *p* = 0.01562; Fig. 7*C*). This finding is consistent with the previous study showing that resurgent sodium current I_NaR_ exists in granule cells (Castelli et al., 2007). Although the future study using whole-bouton recordings of ADP is clearly needed, we suppose that ADP in the mossy fiber terminals is mediated at least partly by voltage-dependent sodium channels, most likely I_NaR_, and causes use-dependent depression of axonal spikes by cumulative inactivation of I_NaT_ by sustained depolarization during ADP.

### The effect of 4-aminopyridine (4-AP) on the PPD of axonal spike

**Figure 8.**
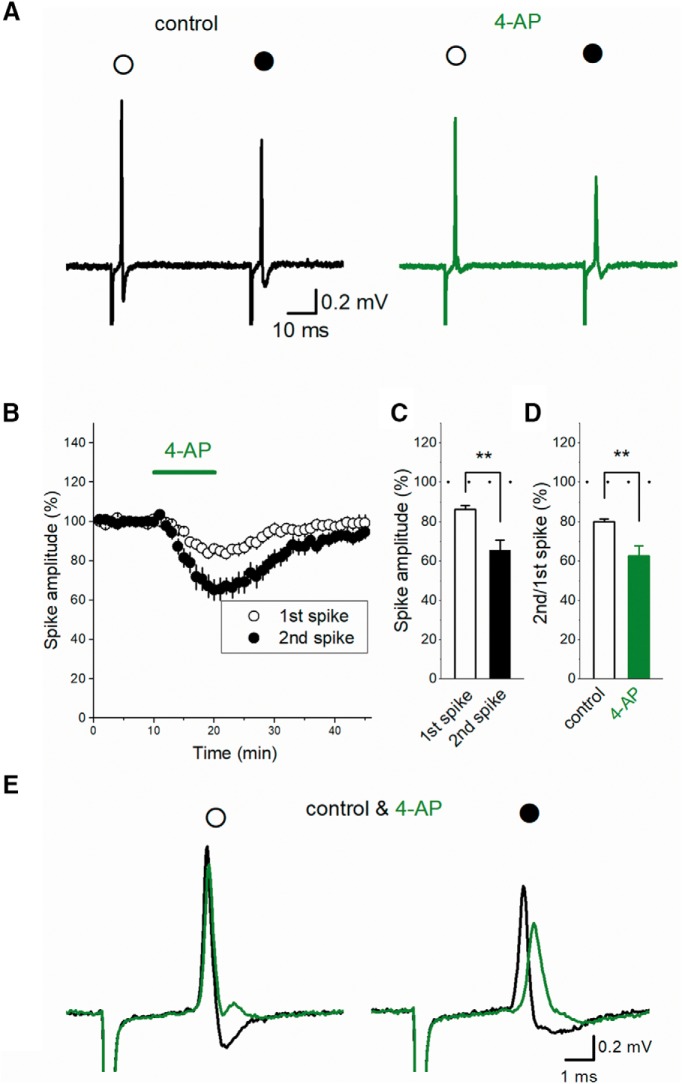
Effect of low concentration of 4-AP on axonal spike. ***A***, 4-AP at 10 μM partially suppressed both the first (open circle) and second spike (closed circle). ***B***, Time course of the amplitude of the first and 2nd spikes during 4-AP application (*n* = 9). ***C***, Summary data of the effect of 4-AP on the first (open bar) and second spikes (closed bar). ***D***, PPD was enhanced by 4-AP (closed bar, *n* = 9, **p < 0.01). ***E***, Superimposed traces of the first (open circle) and second spike (closed circle) in control condition and in the presence of 4-AP. Note that the negative peak of axonal spike was strongly suppressed while the positive peak was weakly affected, suggesting 4-AP may slow the decaying phase of axonal action potentials.

Suppose that cumulative inactivation of I_NaT_ underlies depression of axonal spikes, blocking potassium channels may also modulate subsequent spikes by enhanced inactivation of I_NaT_ during the prolonged initial spike. To check for the specificity of involvement of sodium channels, we tested the effect of potassium channel blocker in PPD of axonal spikes. For this purpose, we adopted low concentration of 4-AP, since action potentials in the mossy fiber terminal have been shown to broaden by slowing repolarization (Carta et al., 2014). Application of 10 μM 4-AP weakly reduced the amplitude of the first spikes to 86.1 ± 2.0%, while the second spikes was strongly suppressed to 65.3 ± 5.2% (*n* = 9, *p* = 0.003906; Fig. 8*A–C*). As a consequence, PPD of axonal spikes (to 80.3 ± 1.8%, *n* = 9) was enhanced by 4-AP (to 61.6 ± 4.6%, *p* = 0.003906; Fig. 8*D*) as similar to veratridine. It should be noted, however, that 4-AP strongly suppressed the negative deflection of axonal spikes (Fig. 8*A*,*E*), as expected from slowing of action potential repolarization. These findings suggest that 4-AP enhances PPD in different ways from veratridine.

## Discussion

In this study, we examined the possible activity-dependent tuning of axonal spikes recorded from single mossy fiber boutons in mouse hippocampal slices. We found that the axonal spikes display robust use-dependent depression in the physiologic frequency ranges or even in the paired-stimuli condition. Notably, veratridine, an inhibitor of inactivation of Na^+^ channels, prominently accelerated use-dependent depression with minimal effect on the first responses. All the results support that sodium channel-dependent mechanisms underlie modulation of use-dependent depression of axonal spikes at the mossy fibers.

### Short-term depression of axonal spikes

Spike propagation along axons is highly reliable digital process which enables reliable information signaling in the central nervous system (Debanne et al., 2011). Recent studies, however, suggested that axonal spikes are subject to analog modulation (Alle and Geiger, 2008; Debanne et al., 2013; Ohura and Kamiya, 2016). At hippocampal mossy fibers, one of the best studied axons in the central nervous system, activity-dependent broadening of action potentials during high-frequency stimulation was reported (Geiger and Jonas, 2000). It was suggested that accumulated inactivation of voltage-dependent K^+^ channels may slow repolarization phase of action potential and thereby prolong the spike duration.

In this study, we found that the amplitude of axonal spikes recorded extracellularly decreased in an activity-dependent manners. PPD of spike amplitude lasted for several hundreds of ms (Fig. 2). Since this time course closely related to that of ADP following action potentials at the hippocampal mossy fibers (Geiger and Jonas, 2000; see also Kamiya et al., 2002), we supposed that ADP may involve in the PPD of axonal spikes. Prolonged-depolarization during ADP may inactivate a fraction of Na^+^ channels, thereby suppress the subsequent action potentials. Consistent with the prediction, delay in the peak of axonal spikes became prominent during repetitive stimuli (Fig. 5*E*).

Detailed kinetic properties of Na^+^ channels at the mossy fiber terminals were examined previously (Engel and Jonas, 2005). They showed that recovery from inactivation was substantially faster than that on the soma, and almost fully recovered within 20 ms. Therefore, remaining inactivation of Na^+^ channels by preceding action potential might not be enough to cause PPD at 50-ms intervals, and we suppose that some additional mechanisms, such as inactivation of Na^+^ channels due to slow ADP, may underlie depression of axonal spikes.

In this study, we adopted Ca^2+^-free conditions to exclude the possibility of recordings of postsynaptic spikes. We wondered if this condition might affect depression of axonal spikes. Therefore, we tested the effect of focal application of Ca^2+^-containing solution to the recording sites, and confirmed that depression was unchanged by this treatment (Fig. 3*D*).

It has been demonstrated that action potentials directly recorded from axons of cerebellar Purkinje neurons in cultures show depression of peak and occasionally conduction failure occurs during repetitive stimulation (Kawaguchi and Sakaba, 2015). Alterations of spike amplitude and potential involvement in failures have also been recorded in Purkinje cell axons in slices (Khaliq and Raman, 2005; Rudolph et al., 2011). In sharp contrast, axonal spikes are resistant to use-dependent depression at the calyx of Held axon terminals which is specialized for high-fidelity signaling even at high-frequency ranges (Sierksma and Borst, 2017). These results suggest that frequency-dependent tuning of axonal excitability may be optimized for types of neuronal signals carried by various sorts of axon.

### Sodium channel-dependent modulation of short-term depression

It was reported that veratridine, an inhibitor of inactivation of Na^+^ channels, robustly upregulates ADP, but not action potentials themselves, at the calyx of Held presynaptic terminals (Kim et al., 2010). We therefore examined whether veratridine may modulate depression of axonal spikes without affecting the initial action potentials. Consistent with the prediction, veratridine accelerated short-term depression of axonal spikes, whereas the amplitude of the initial spikes was almost unaffected.

Voltage-gated sodium channels are categorized into three subtypes with different mode of activation, namely the transient type (I_NaT_), the persistent type (I_NaP_), and resurgent type (I_NaR_). The I_NaT_ is essential for generation of action potential, although I_NaP_ and I_NaR_ are supposed to increase neuronal excitability and regulate burst firings (Raman and Bean, 1997; D'Ascenzo et al., 2009; Kole, 2011). Since veratridine is a rather non-specific inhibitor of inactivation of sodium channels, it may exert facilitative actions on all of I_NaT_, I_NaP_, and I_NaR_.

Involvement of sodium channels was also suggested by the findings as below. A low concentration of sodium channel blocker TTX at 50 nM slightly, but significantly restored PPD of axonal spikes, as shown in Figure 6. We also tested for 20 nM TTX, which was reported to suppress ADP at the calyx of Held axon terminals (Kim et al., 2010). TTX at 20 nM weakly suppressed the amplitude of first spike, although PPD of axonal spikes was not affected significantly. We could not explain the reason why PPD was not affected by 20 nM TTX significantly. The difference in preparation (e.g., calyx of Held versus mossy fiber axon terminals) or experimental conditions may explain the minimal effect of 20 nM TTX. Alternatively, additional mechanisms independent of activation of sodium channels, e.g., capacitative components of propagating action potentials may mediate ADP at least in part (Kim et al., 2010).

On the other hand, potassium channel blocker 4-AP also enhanced PPD as veratridine, although the wave form of axonal spikes was changed significantly, as expected from slowing of repolarization by 4-AP (Carta et al., 2014). Taken together, our results suggest the involvement of sodium channels, rather than potassium channels, in ADP and short-term depression of axonal spikes.

To account for the selective effects on short-term depression of axonal spikes with minimal effect on initial action potential, we considered as follows. Hippocampal mossy fiber terminals express high-density of Na^+^ channels which amplify action potentials (Engel and Jonas, 2005), and secure safe conduction over multiple boutons which are expected to be a risk factor for conduction failure due to impedance mismatch of thin axons and large boutons. Highly abundant expression of sodium channels suggests that a fractional enhancement of I_NaT_ may not modify the amplitude of action potentials caused by regenerative processes substantially. In other words, the facilitative effect of veratridine on I_NaT_, if any, may not become obvious on the peak amplitude of action potentials. In any case, the selective modulation by veratridine revealed sodium channel-dependent mechanisms, possibly through enhancing I_NaP_ or I_NaR_, underlay short-term depression of axonal spikes.

In this study, our observations were limited in loose-patch clamp recordings, since they are less invasive and stable for prolonged periods, and thereby it was possible to show whole time course of the veratridine effect including washout. In support of our interpretation, ADP recorded from the granule cells in dentate gyrus was shown to be enhanced and prolonged by veratridine (Fig. 7). Since granule cells express resurgent sodium current I_NaR_ (Castelli et al., 2007), veratridine activated I_NaR_ and enhanced ADP. Similar mechanisms in the mossy fiber terminals may account for the accelerated depression of axonal spike observed in this study. To directly assess the mechanisms, however, whole-bouton recording would help in getting the mechanistic insights.

Veratridine robustly enhanced and prolonged ADP recorded from the granule cells in the dentate gyrus, and caused multiple spiking by single stimuli (Fig. 7). It was speculated that much stronger effect of veratridine on somatic ADP might be resulted either from different modes of AP stimulation or different recording configurations (i.e., whole-cell recordings versus non-invasive cell attached recordings).

It would be also helpful to perform numerical simulations by modeling study. To date, several realistic models of action potentials have been proposed in hippocampal mossy fibers (Engel and Jonas, 2005; Alle et al., 2009; Schmidt-Hieber and Bischofberger, 2010). All these previous simulations do not incorporate ADP in their models. It is obvious that the revised model incorporating ADP after identifying the ionic mechanisms by future experiments.

### Subcellular recordings from axon terminals

In this study, we established direct recordings from the single axon terminals of the hippocampal mossy fibers, following stimulation of their originating soma (granule cells in dentate gyrus). This approach will offer unique opportunities to study spike signaling along axons, since hippocampal mossy fibers consist of *en passant* structures typical for many cortical axons. It should be mentioned that recent studies clarified many important notions on the heterogeneity of ionic conductances (Kole and Stuart, 2012; Debanne et al., 2013) as well as local control by the influence of microenvironment. Ectopic spiking of axon terminals (Dugladze et al., 2012) may be the important subject of future studies.

### Functional implications

Despite functional importance, use-dependent short-term plasticity of spike signaling has not been studied intensively. Slight but reproducible depression even at paired-pulse conditions, as observed in this study, suggest the physiologic significance of this form of analog tuning. Since modification of axonal spikes has strong impact on transmitter release and plasticity by affecting entry of Ca^2+^ into the presynaptic terminals (Geiger and Jonas, 2000; Kawaguchi and Sakaba, 2015), we suggest that short-term depression of axonal spikes may play an important modulatory role in short-term plasticity at the mossy fiber-CA3 synapses. Further studies with whole-bouton recordings will clarify the detailed mechanisms of this novel presynaptic form of plasticity.

## Synthesis

Reviewing Editor: Pablo Castillo, Albert Einstein College of Medicine Decisions are customarily a result of the Reviewing Editor and the peer reviewers coming together and discussing their recommendations until a consensus is reached. When revisions are invited, a fact-based synthesis statement explaining their decision and outlining what is needed to prepare a revision will be listed below. The following reviewer(s) agreed to reveal their identity: Steven Mennerick, Linda Overstreet-Wadiche.

The reviewers agree in that you have address most of your concerns but they also indicate you must edit your manuscript. Note that Reviewer #1 requests further analysis and Reviewer #2 requests a new experiment that you should perform. I strongly recommend that you thoroughly address all of their comments.

-----------------------------------------------------------------

REVIEWER #1

I still feel the authors are guilty of some over interpretation, but the previous requests have mostly been addressed.

One major concern not addressed in the rewrite is the interpretation of the extracellular waveforms. The authors note that the extracellular waveforms are proportional to the first derivative of Vm, but they repeatedly refer to the change in recorded extracellular waveform as a change in the amplitude of the action potential. The impact on the action potential peak and the nature of the after polarization are not clear without further analysis (e.g., reconstruction of the intracellular AP from the extracellular waveform). It is likely the maximum slope and perhaps half-width (not peak) that are most affected. This deserves some treatment in the discussion.

Minor

Lines 305-308, awkward wording. Suggest ‘during a 20 Hz train was prolonged relative to the 1st spike...’ Also, please clarify: latency to onset or latency to peak?

Line 371-2 and elsewhere, I think it is more correct to talk/write of veratridine as an inhibitor of Nav inactivation rather than Nav ‘opener.’ An opener would predict a stronger effect on first spikes than observed.

Line 309-310. See major point above. What is the significance/interpretation of a widening of the dV/dt signal?

Line 460: awkward construction. Suggest ‘In support of our interpretation.’

REVIEWER #2

The authors addressed the concerns with additional experiments, as requested. One issue that would have more strongly supported the proposed mechanism (Line 321) would have been to show that 50 nM TTX reduced the ADP in Fig 7, and to test a lower concentration of TTX that did not affect the first spike.

Minor comments:

Line 101. The university should be inserted into the empty field.

Line 231. Perhaps this could be explained more clearly, something like “To confirm spike recordings from axon terminals, we used Ca2+ free solution to exclude the possibility of synaptically evoked spikes in postsynaptic cells”.

Line 306 & 311. “The latency of 10th spike during 20 Hz train was prolonged than the 1st spike”. Should be “more than” or “compared to”

Fig. 7. There is a very robust effect of veratridine on the whole-cell ADP that appears stronger than the effect on axonal spikes (i.e. multiple spikes). The authors should speculate on this difference, which could include lack of soma in MF terminal recordings, different modes of AP stimulation or whole cell vs. non-invasive cell attached recording modes.

Line 414. Alterations of spike amplitude and potential involvement in failures have also been recorded in Purkinje cell axons in slices (i.e. Khaliq and Raman J Neuro 2005; Rudolph et al., Neuron, 2011). This paragraph ends this discussion section somewhat abruptly - it might be useful if the authors include a summary statement about how their results integrate with the other literature discussed in this section.
